# Supplementary figures and images for: Cellular components in tumor microenvironment of neuroblastoma and the prognostic value
Source: PeerJ. 2019 Dec 10;7:e8017. doi: 10.7717/peerj.8017 (PMC6910112; doi:10.7717/peerj.8017)

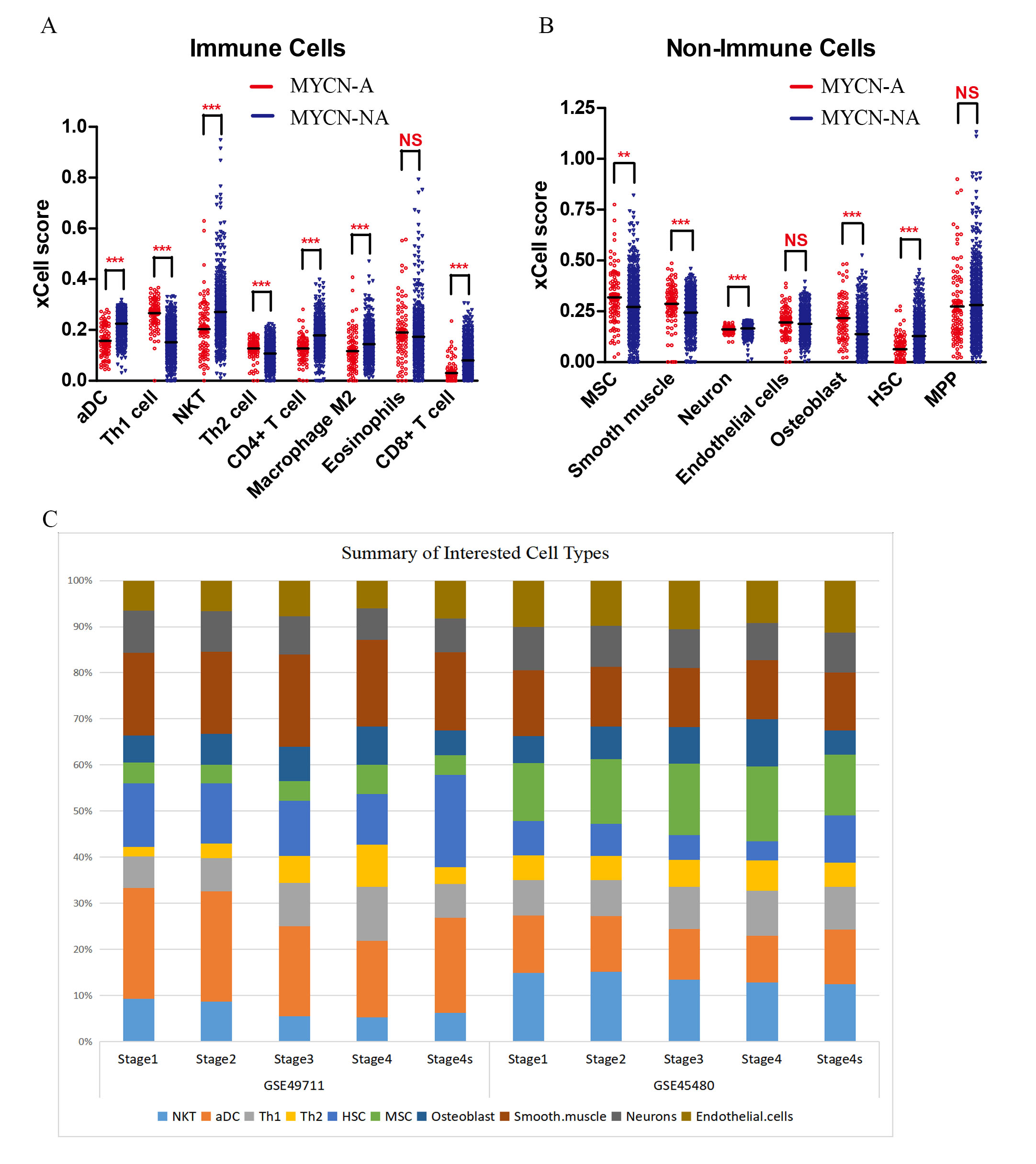

Supplement: Figure S1 — (A, B) xCell score of immune and non-immune cells in GES45480; (C) Summary of inferred cell subtypes in INSS stage 1-4s in GSE49711 and GSE45480. [file peerj-07-8017-s006.jpg]

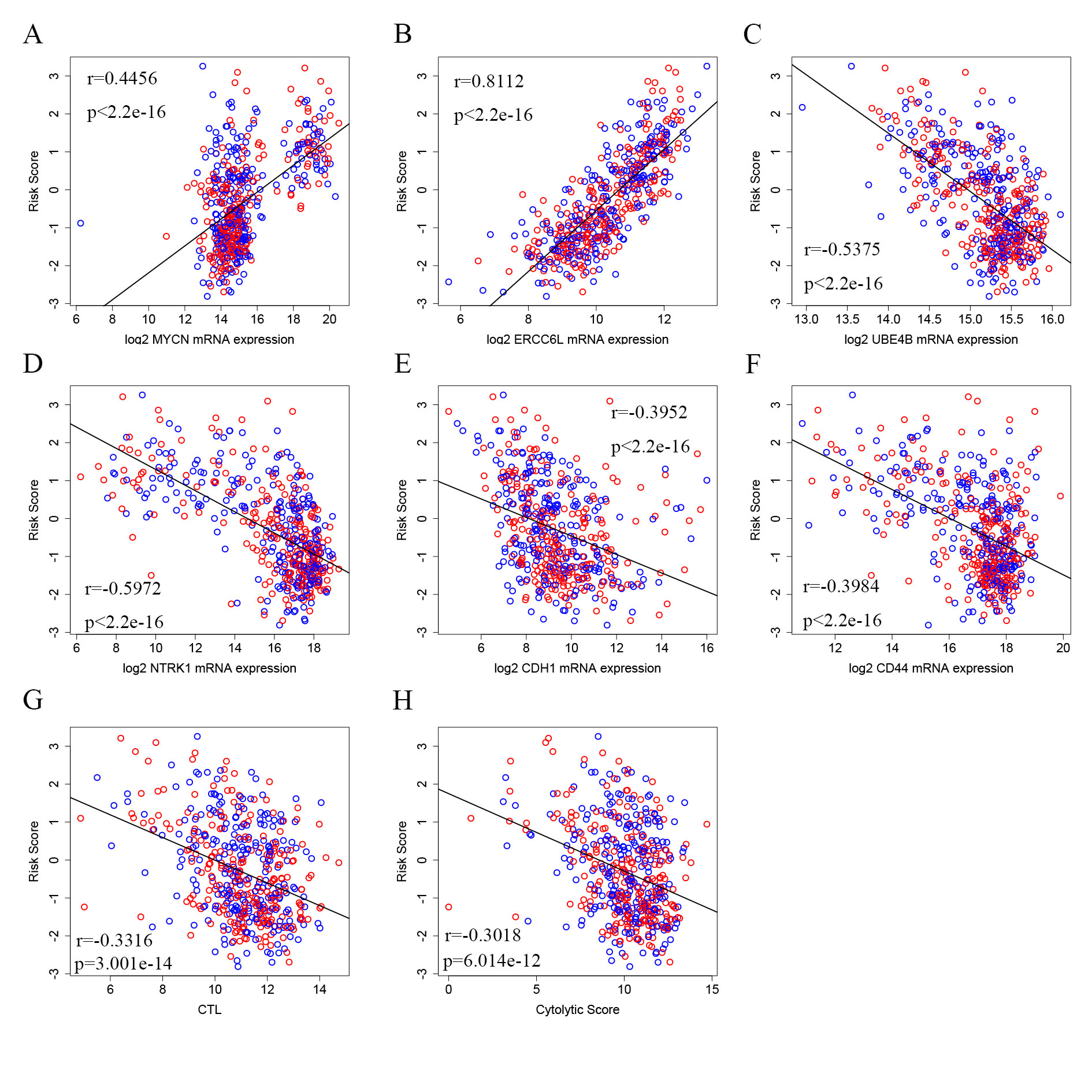

Supplement: Figure S2 — (A)Spearman correlation of pCRS with MYCN mRNA expression. (B) Spearman correlation of pCRS with ERCC6L mRNA expression. (C–F) Spearman correlation of pCRS with UBE4B, NTRK1, CDH1, and CD44 mRNA expression. (G, H) Spearman correlation of pCRS with immune marker CTL and CYT. pCRS: prognostic cell risk score. [file peerj-07-8017-s007.jpg]
